# Effect of acute kidney injury care bundle on kidney outcomes in cardiac patients receiving critical care: a systematic review and meta-analysis

**DOI:** 10.1186/s12882-025-03955-1

**Published:** 2025-01-10

**Authors:** Fatma Refaat Ahmed, Nabeel Al-Yateem, Seyed Aria Nejadghaderi, Rawia Gamil, Mohannad Eid AbuRuz

**Affiliations:** 1 College of Health Sciences, Department of Nursing, University of Sharjah, Sharjah, UAE; 2 Critical Care and Emergency Nursing Department, Faculty of Nursing, Alexandria University, Alexandria, Egypt; 3 HIV/STI Surveillance Research Center, and WHO Collaborating Center for HIV Surveillance, Institute for Futures Studies in Health, Kerman University of Medical Sciences, Kerman, Iran; 4 Systematic Review and Meta-analysis Expert Group (SRMEG), Universal Scientific Education and Research Network (USERN), Tehran, Iran; 5 College of Nursing and Midwifery, MBRU, Dubai Health, Dubai, UAE

**Keywords:** Care Bundle, Acute renal failure, Critical care, Intensive care unit, Cardiac Disease, Systematics Review, Meta-analysis

## Abstract

**Background:**

Cardiac surgery is a major contributor to acute kidney injury (AKI); approximately 22% of patients who undergo cardiac surgery develop AKI, and among them, 2% will require renal replacement therapy (RRT). AKI is also associated with heightened risks of mortality and morbidity, longer intensive care stays, and increased treatment costs. Due to the challenges of treating AKI, prevention through the use of care bundles is suggested as an effective approach. This review aimed to assess the impact of care bundles on kidney outcomes, mortality, and hospital stay for cardiac patients in critical care.

**Methods:**

PubMed, Scopus, Web of Science, and EMBASE were searched up to November 2024. Inclusion criteria were studies on individuals with cardiac diseases receiving critical care, that used AKI care bundle as the intervention, and reported outcomes related to AKI, mortality, and other kidney-related events. We used the Cochrane Collaboration’s risk of bias tool 2 and the Newcastle-Ottawa scale for quality assessment. Pooled odds ratios (ORs) or risk ratios (RRs) with 95% confidence intervals (CIs) were calculated.

**Results:**

Seven studies on total 5045 subjects, including five observational and two randomized controlled trials (RCTs) were included. The implementation of care bundles significantly reduced the incidence of all-stage AKI (OR: 0.78; 95%CI: 0.61–0.99) and moderate-severe AKI (OR: 0.56; 95%CI: 0.43–0.72). Also, the implementation of care bundle increased the incidence of persistent renal dysfunction after 30 days by 2.39 times. However, there were no significant changes in RRT, major adverse kidney events, or mortality between the groups. The mean quality assessment score for observational studies was 7.2 out of ten, while there were noted concerns in the risk of bias assessment of the RCTs.

**Conclusions:**

The application of care bundles in patients, including those undergoing cardiac surgeries as well as non-cardiac critical illness, appears to be effective in reducing AKI, particularly in moderate and severe stages. However, given the inclusion of non-cardiac patients in some studies, the observed effect may not be solely attributable to cardiac surgery cases. Future large-scale RCTs focusing specifically on cardiac surgery patients are recommended to clarify the impact of care bundles within this subgroup.

**Registration ID in PROSPERO:**

CRD42024498972.

**Supplementary Information:**

The online version contains supplementary material available at 10.1186/s12882-025-03955-1.

## Introduction

Cardiac surgery is a major correlate of acute kidney injury (AKI) development. However, AKI can also develop in cardiac patients who do not undergo surgical intervention, and this population warrants specific focus. In this regard, the global incidence rate of AKI among those undergoing cardiac surgeries is 22.3%, and 2.3% of patients require renal replacement therapy (RRT) [1]. Moreover, AKI in cardiac patients is linked to increased risks of mortality and morbidity. Specifically, post-cardiac surgery patients who develop AKI have a 68% higher risk of long-term mortality [2]. In addition, AKI in these patients is associated with longer intensive care unit (ICU) stays and a higher incidence of atrial fibrillation [3]. The overall costs of AKI per patient vary widely, ranging from $29,700 for those undergoing cardiac surgery to $89,400 for patients experiencing cardiogenic shock [4].

Several risk factors for the development of AKI among individuals hospitalized in ICUs have been suggested, including advanced age, comorbidities like diabetes, heart failure, and hypertension, elevated baseline creatinine levels, nephrotoxic drug use, vasopressor/inotrope use, high-risk surgeries, and extended periods on the cardiopulmonary bypass pump [5]. Because of the difficulty of AKI treatment, preventive measures such as adequate renal blood flow and preventing exposure to adverse renal substances, including nephrotoxic drugs and insults related to blood transfusions are recommended [6]. To address this, a structured method involving a combination of several measurements and evidence-based practices to improve patient outcomes is implemented through care bundles such as the Enhanced Recovery After Cardiac Surgery (ERACS) protocol and the Kidney Disease Improving Global Outcomes (KDIGO) guidelines [7]. The ERACS protocol encompasses a coordinated series of perioperative strategies aimed at reducing the physiological stress of surgery and supporting faster recovery to preoperative function. Implementing these bundles, especially ERACS, requires significant interdisciplinary coordination, patient engagement, and team training to ensure each step is carried out effectively [8]. The KDIGO care bundle is one of the examples that recommends preventing the use of nephrotoxic substances, temporarily holding angiotensin-converting enzyme inhibitors and angiotensin II receptor blockers within the initial 48 h post-surgery, closely tracking serum creatinine levels and urine output, avoiding high blood sugar levels during the initial 72 h post-surgery, exploring alternatives to radiocontrast agents, and employing close hemodynamic monitoring [9]. Effective outcomes with care bundles necessitate comprehensive training and education of the nursing teams. Moreover, health policymakers should understand the personal and economic impact arising from AKI to provide support for commissioning, improvement methodologies, and registry initiatives, along with research investments for sustaining advancements and the overall management [10].

While previous meta-analyses have evaluated AKI care bundles, they have largely focused on general populations or non-cardiac surgery patients, overlooking this key subset of patients. A prior systematic review and meta-analysis evaluated the effects of compliance with the AKI care bundle and outcomes of hospitalized patients [11]. However, it did not focus on patients with cardiac disease and those hospitalized in ICUs. Furthermore, it only searched three databases, and the search date was up to June 2021. Also, it did not provide baseline characteristics of the included studies [11]. Accordingly, another systematic review evaluated the efficacy of the AKI care bundle with or without the use of biomarkers on kidney outcomes [12]. Similarly, it did not specifically include patients with cardiac disease receiving critical care. Given that both cardiac surgery and critical cardiac conditions are major factors for AKI development, and with the publication of several primary studies in recent years, there is a need to evaluate the roles of AKI care bundle in prevention and outcomes for those undergoing cardiac surgeries or with cardiac diseases. Herein, our objective was to investigate the effects of care bundles on outcomes of AKI, mortality, and length of hospital or ICU stay in cardiac patients receiving critical care compared to those who did not receive care bundle measures.

## Methods

The study was conducted and reported in adherence to the Preferred Reporting Items for Systematic Reviews and Meta-Analyses (PRISMA) 2020 guidelines [13]. We submitted the protocol of the systematic review in the International Prospective Register of Systematic Reviews (PROSPERO) (Registration ID: CRD42024498972).

### Database searching

Databases including PubMed, Scopus, Web of Science, and EMBASE were systematically searched from inception to December 27, 2023. The search was updated on November 9, 2024. A combination of terms related to three concepts which were “Cardiovascular disease”, “patient care bundle”, and “acute kidney injury” were used. Some of the terms were (“Cardiovascular Diseases” OR “Heart Diseases” OR “Myocardial Ischemia” OR “Myocardial Infarction” OR “cardiac event” OR “Ischemic Heart Disease” OR “coronary disease”) AND (“Patient Care Bundles” OR “Evidence-Based Practice” OR “Clinical bundle” OR “Protocol bundle” OR “Intervention bundle” OR “Evidence-based care” OR “Evidence Based Practice”) AND (“Acute Kidney Injury” OR “Renal Dialysis” OR “Renal Replacement Therapy” OR “Acute Renal Insufficiency” OR “Acute Renal Failure”) (Table S1).

There were no limitations on search fields, such as language, date, or study types. We used both backward (i.e., reviewing the citations or references of an article) and forward citation searches (i.e., a search aimed at identifying all articles that reference a particular article). As the grey literature search, the initial 300 records retrieved from the Google Scholar search engine were evaluated [14]. Moreover, we searched the clinicaltrials.gov website for any other potential eligible clinical trials (Table S1).

### Study selection

In this review, the inclusion criteria required studies to involve individuals with various cardiac diseases, such as myocardial infarction, who were receiving critical care. The intervention group must have received an AKI care bundle, as defined by relevant national or international guidelines. If a comparison group was present, it must have received routine, standard, or alternative care, excluding the defined AKI care bundle. Studies had to report outcomes related to AKI, including the occurrence of AKI, in-hospital mortality, length of hospital or ICU stay, kidney replacement therapy, hemodialysis, persistent renal dysfunction (PRD) defined as a sustained increase in serum creatinine levels ≥ 0.5 mg/dl compared to baseline, or major adverse kidney events (MAKE). Additionally, studies had to provide either effect sizes or raw data for outcomes, allowing for the calculation of effect sizes. We included studies conducted in any country and published in any language, as long as they met these criteria. Eligible study designs included clinical trials, cohort studies, case-control studies, and cross-sectional studies.

The exclusion criteria applied to studies that involved participants without cardiac diseases or those who were not admitted to the ICU or did not receive critical care. Studies involving participants who had AKI at baseline or those that did not receive an AKI care bundle were also excluded. Additionally, we excluded case reports, review articles, editorials, meta-analyses, letters, conference proceedings, protocols, and in-vitro or animal studies. Studies without a proper comparison group, or where the comparison group did not fit the criteria outlined in this review, were also excluded.

The primary endpoint of this review was the occurrence and severity of AKI, including whether patients required kidney replacement therapy or hemodialysis. Secondary endpoints included the in-hospital mortality rate, the length of hospital or ICU stay, PRD as defined by a sustained increase in serum creatinine ≥ 0.5 mg/dl compared to baseline, and MAKE.

Management and deduplication of references were conducted in EndNote software. First, the title and abstract of articles were screened. Then, the full-texts of the included articles were assessed according to the eligibility criteria. They were conducted by one of the reviewers (SAN) under the supervision of the principal investigator (FRA). We contacted the corresponding authors of studies that we could not find their full-texts three times with a one week interval.

### Data extraction

We extracted the following data: (1) Baseline characteristics (first author name, country, study design, year, study population, and sample size); (2) Characteristics of participants (age, sex, race/ethnicity, follow-up, comorbidities, and type of surgery); (3) Intervention and control characteristics (type of care bundle and control); (4) relevant data for outcomes of interest for qualitative or quantitative synthesis; and (5) other information like compliance to care bundle or definition of outcomes. The data extraction was done in a spreadsheet in Microsoft Office Excel. We consulted the principal investigator (FRA) in case of any discrepancy.

### Quality assessment

We used the Cochrane Collaboration’s risk of bias tool 2 (RoB 2) for randomized controlled trials (RCTs) [15] and the Newcastle-Ottawa scale for observational studies [16]. The quality assessment was performed by one reviewer (SAN) and the principal investigator (FRA) was contacted in case of any uncertainty.

### Statistical analysis

We used Stata 17.0 (Stata Corp, LLC, TX) for quality assessment. We reported the pooled odds ratio (OR) or risk ratios (RRs) with 95% confidence intervals (CIs) for dichotomous data using either random or fixed-effects models. The decision to use the random-effects model was influenced by the perceived methodological heterogeneity of the actual effect sizes. For this, Cochran’s Q and the I-square statistic were used to assess between-study heterogeneity. The I² statistic represents the percentage of total variation across studies due to heterogeneity rather than chance. An I-square value greater than 50% was considered as substantial heterogeneity and the random-effect model was used [17]. The heterogeneity measure τ² was calculated using a random-effects model, with its 95% confidence intervals estimated using the method described by DerSimonian and Laird. For continuous outcomes (i.e., length of hospital and ICU stay), we reported pooled mean difference with 95% CIs. The median and interquartile range (IQR) were converted to mean and standard deviation, according to the article by Abbas et al. [18]. Assessment of publication bias was conducted through the Egger’s test, where a significance level of *p* < 0.05 was considered as an indication of statistically significant publication bias [19]. Given that the Egger’s test may lack reliability with fewer than ten studies, we also inspected the forest plot visually for asymmetry as a supplementary assessment of publication bias. Although funnel plot asymmetry is typically recommended for this purpose, it is considered less reliable with small samples; thus, a forest plot inspection was used to observe potential patterns in effect sizes that could suggest bias. Given the limited number of included studies, we conducted a sensitivity analysis using the Hartung-Knapp-Sidik-Jonkman (HKSJ) method. This approach is particularly useful for small meta-analyses as it adjusts for the potential bias in variance estimation in random-effects models, providing more reliable results when the number of studies is small. We performed this analysis for the main outcomes to ensure the robustness of the findings [20]. For studies without a comparison group, we used a narrative synthesis to report the findings.

## Results

**Fig. 1:**
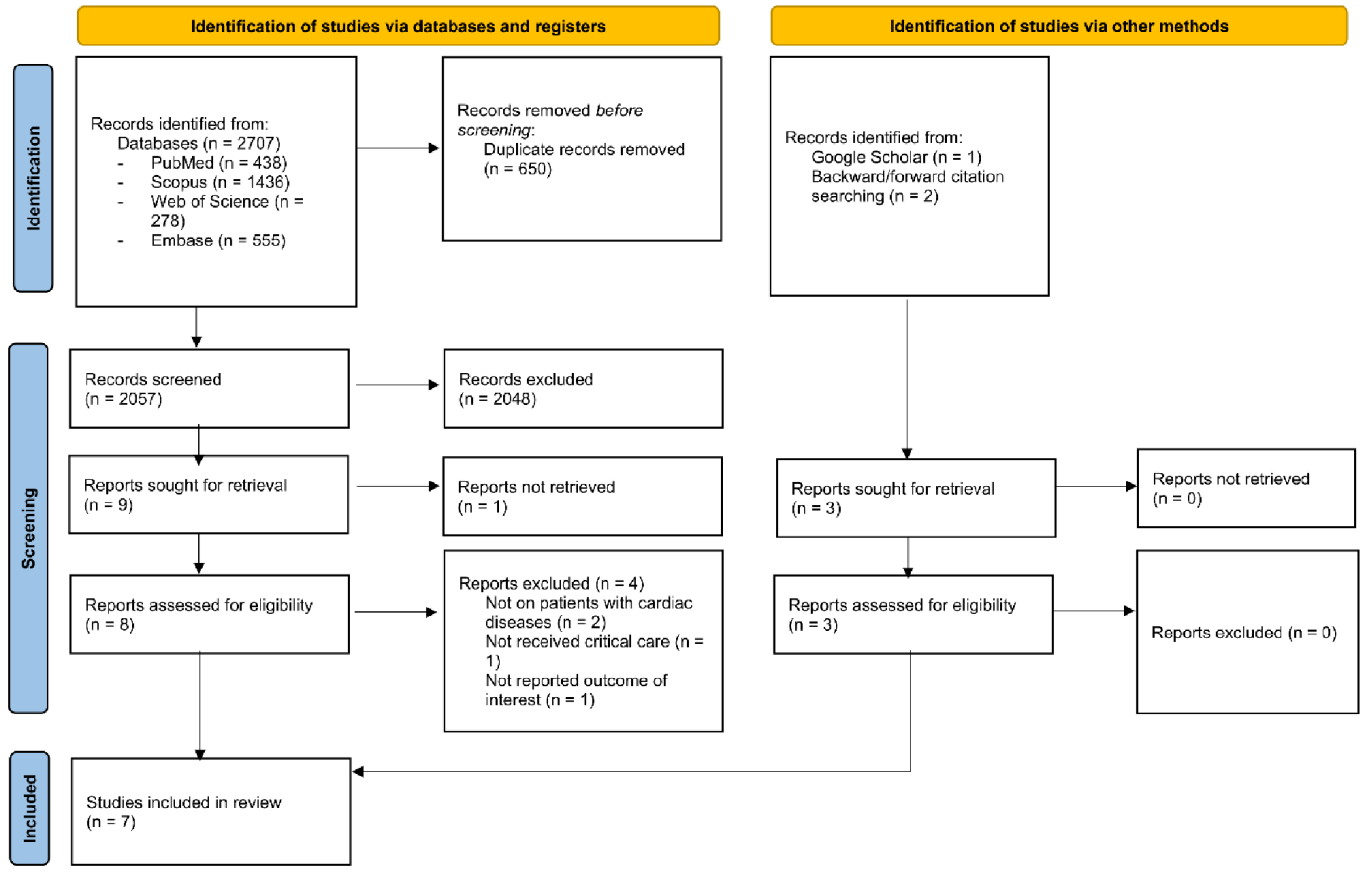
Study selection process

In the updated search, we found a total of 2707 records from the database searching. Following the duplicate removal, there were 2057 for the title/abstract screening. In this step, 2048 records were excluded. Then, we should retrieve the full-texts of nine studies. We could not find the full-text of one study [21]. Out of eight studies in the full-text reviewing, we excluded four studies in which two did not involve patients with cardiac diseases [22, 23], one did not report the outcomes of interest [24], and in one study the participants did not receive critical care [25]. Four studies were included in this step [26–29]. We also found two other eligible studies in citation searching [30, 31] and one in searching Google Scholar [32]. Finally, seven studies were included [26–32] (Fig. 1).

### Study characteristics

Out of seven studies, five were observational studies [28–32] and two were open-label parallel-group RCTs [26, 27]. Two studies were multicentral from Europe [26, 27], and one study conducted in each countries of United Kingdom [32], Germany [26], United States [30], Belgium [31], and Israel [28]. All of the studies involved adult participants undergoing cardiac surgery. Two studies followed the patients up to 30 days [29, 31], two studies up to 90 days [26, 27], and one study had a follow-up duration of 48 months [28]. Five studies used the KDIGO criteria for the definition of AKI and AKI severity [26–30], one study defined AKI as a raised creatinine level of more than 30% from the preoperative level [32], and one defined AKI severity based on the AKI network definition [31]. Four studies used KDIGO bundle as the intervention [26, 27, 29, 30], two used Enhanced Recovery After Cardiac Surgery (ERACS) [31, 32], and one used AKI care bundle [28]. Table S2 describes the elements of each care bundle guideline. As the comparison, five studies used routine or standard care before the implementation of the care bundle [26–28, 30, 32] and two studies did not have a control group [29, 31] (Table 1).

**Table 1:** Characteristics of included studies

Study ID	Country	Study design	Study population	AKI definition	Number of participants	Number (%) of males	Mean (SD) or median (IQR) for age (year)	Comorbidities	Surgery type	Care bundle	Definition of control
Fleming et al. 2016	UK	Prospective observational	Patients undergoing cardiac surgery during October 2010 before and during July 2011 after implementation of the ERACS protocol	Raised creatinine level of more than 30% from the preoperative level	Total: 105; Case: 52; Ctrl: 53	Total: 76 (72.38%); Case: 38 (73.1%); Ctrl: 38 (71.7%)	Case: 68.6 (11.1); Ctrl: 66.5 (11.8)	HF, angina pectoralis, hypertension, previous MI, COPD, PONV, hypercholesterolemia	CABG, CABG + AVR, CABG + MVR, AVR, MVR	ERACS with preoperative and postoperative bundles	Patients undergoing cardiac surgery before implementation of the ERACS protocol
Meersch et al. 2017	Germany	Open-label parallel-group RCT	Adults at high risk for AKI who underwent cardiac surgery with the use of CPB at the University of Muenster, Germany, between August 2014 and December 2015	KDIGO criteria	Total: 276; Int: 138; Ctrl: 138	Total: 199 (72.1%); Int: 94 (68.1%); Ctrl: 105 (76.1%)	Int: 68.4 (11.2); Ctrl: 68.3 (11.6)	Hypertension, diabetes, COPD, CKD, previous heart surgery, MI, AF	CABG, valve, CABG + valve, other	KDIGO CT surgery bundle	Standard care
Engelman et al. 2020	US	Retrospective observational	Adults undergoing a cardiac operation with CPB between July 2016 and June 2018	KDIGO criteria	Total: 847; Case: 412; Ctrl: 435	Total: 613 (72.4%); Case: 311 (75.5%); Ctrl: 302 (69.4%)	Case: 66.3 (10.5); Ctrl: 66.3 (10.6)	Diabetes, dyslipidemia, CLD, hypertension, liver disease, cancer, cerebrovascular disease, sleep apnea, depression, peripheral artery disease, previous MI, cardiac arrhythmia, previous cardiac intervention	CABG, CABG + AVR, CABG + MVR, AVR, MVR, AVR + MVR, MV repair, MV repair + CABG, others	KDIGO cardiac surgery bundle	Standard care
Zarbock et al. 2021	12 centers in Europe (Belgium, Germany, Italy, Spain, and UK)	Open-label parallel-group RCT	Adults undergoing cardiac surgery involving the use of CPB between November 2017 and November 2019	KDIGO criteria	Total: 278; Int: 136; Ctrl: 142	Total: 195 (70.1%); Int: 94 (69.1%); Ctrl: 101 (71.1%)	Int: 66.9 (10.6); Ctrl: 66.0 (10.3)	Hypertension, diabetes, COPD, CKD, previous heart surgery, MI, AF	CABG, Valve, CABG + valve, other	KDIGO bundle	Standard care
Hoogma et al. 2022	Belgium	Retrospective observational	Adults undergoing cardiac surgery at the University Hospitals Leuven and being admitted to the PACU-centric ERACS program between January 1, 2019, and December 31, 2019	AKI network	Total: 356; Case: 356; Ctrl: 0	Total: 258 (72.4%); Case: 258 (72.4%); Ctrl: 0	Case: 66 (57–73)	NA	CABG, valve surgery, full sternotomy, minimally invasive, CPB	ERACS	None
Khoury et al. 2023	Israel	Retrospective observational	Patients with MI admitted following PCI between January 2008 and December 2020	KDIGO criteria	Total: 2646; Case: 705; Ctrl: 1941	Total: 2156 (81.5%); Case: 589 (83.5%); Ctrl: 1567 (80.7%)	Case: 62 (13); Ctrl: 62 (13)	Hypertension, diabetes, hyperlipidemia, anemia	PCI	AKI-CB	Before the AKI–CB implementation
Massoth et al. 2023	Six centers in Germany, Spain, and Russia	Prospective observational	Adults undergoing cardiac surgery with CPB between February and November 2021	KDIGO criteria	Total: 537; Case: 537; Ctrl: 0	Total: 367 (68.3); Case: 367 (68.3); Ctrl: 0	Case: 66 (58.72)	HF, preoperative IABP, COPD, diabetes, previous cardiac surgery	Emergency surgery, CABG, Valve, CABG + valve, other	KDIGO bundle	None

Abbreviations: NA: not available; RCT: randomzied controlled trial; UK: United Kingdom; US: United States; Int: Intervention; Ctrl: Control; SD: standard deviation; IQR: inter-quartile range; ERACS: Enhanced Recovery After Cardiac Surgery; HF: heart failure; IABP: intra-aortic balloon pump; COPD: chronic obstructive pulmonary disease; CABG: coronary artery bypass graft; AKI-CB: acute kidney injury care bundle; KDIGO: Kidney Disease Improving Global Outcomes; PCI: percutaneous intervention; MI: myocardial infarction; PONV: Postoperative nausea and vomiting; CKD: chronic kidney disease; AVR: Aortic Valve Replacement; MVR: Mitral Valve Replacement; AF: atrial fibrillation; CPB: cardiopulmonary bypass; CLD: chronic lung disease; MV: mitral valve; PACU: post-anesthesia care unit

The total number of participants was 5045, with individual study sample sizes ranging from 105 to 2646. Overall, most participants were males (*n* = 3864; 76.59%) and the mean age across studies ranged from 62.0 to 68.6 years. Only two studies reported race/ethnicity, with Caucasian participants constituted the majority [30, 32]. Coronary artery bypass graft (CABG), valve surgeries, or a combination of both were the most commonly reported procedures [26, 27, 29–32], while one study focused on participants undergoing percutaneous coronary intervention [28]. A range of comorbidities, including cardiovascular, respiratory, mental, and endocrine disorders was also reported (Table 1).

### Qualitative synthesis

Two studies did not have control groups or the control group was not those who did not receive care bundle [29, 31]. AKI was occurred in 27.19% of participants in the article by Hoogma et al. [31]. In the same study, the frequencies of 30-day mortality and readmission were 0.8% and 9.0%, respectively [31]. The frequencies of moderate-severe AKI were 3.37% and 8.38% in the articles by Hoogma et al. and Massoth et al. studies, respectively [29, 31]. In the article by Massoth and colleagues, PRD, RRT, 30-day MAKE occurred in 14.3%, 1.1%, and 5.0% of participants, respectively [29].

The length of hospital stay was reported in all studies. The median duration of hospital stay ranged from 6 days [31, 32] to 13 days in the intervention group or cases [29]. The median duration of hospital stay in controls ranged from 6 days [32] to 11 days [26, 27]. The mean duration of hospital stay was 5.2 and 11.2 days among cases and 5.9 and 10.7 days among controls [28, 30].

The study by Massoth et al. did not have a control group. In this study, the median lengths of hospital and ICU stay were 13 (IQR: 10–18) and 2 (IQR: 1–3) days, respectively [29].

Three studies reported the compliance to care bundle [27, 29, 31]. It was 64% in the study by Hoogma et al. [31]. In another one, the compliance to all measures, three, and four/five components were 0.4%, 34.6%, and 16.8%, respectively [29]. In Zarbock et al., it was significantly higher in the intervention group than controls (65.4% vs. 4.2%) [27].

### Quantitative synthesis

#### All-stage AKI and stage 2–3 AKI

**Fig. 2:**
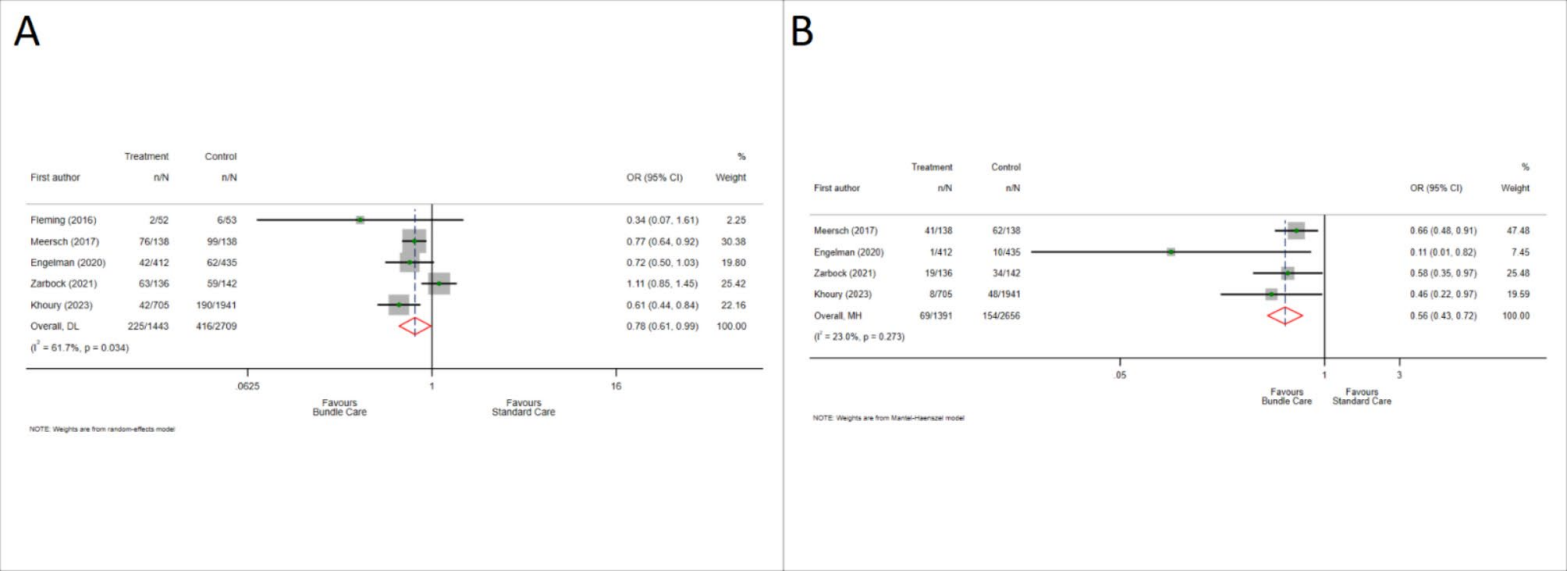
Forest plot of the association between receiving care bundle compared to standard care and the outcomes of acute kidney injury (AKI) (**A**) and stage 2 and 3 AKI (**B**) in participants with cardiac diseases. OR: odds ratio; CI: confidence interval

Five and four studies reported the occurrence of all-stage AKI and stage 2–3 AKI, respectively. The use of care bundle significantly reduced the incidence of all stage AKI (OR: 0.78; 95% CI: 0.61, 0.99) (Fig. 2A) and stage 2–3 AKI (OR: 0.56; 95% CI: 0.43, 0.72) (Fig. 2B). There was a significant publication bias according to Egger’s test for stage 2–3 AKI (*p* = 0.03), however, no significant publication bias was found for all-stage AKI (*p* = 0.77).

#### RRT

Two RCTs reported the incidence of RRT. The use of care bundle did not change the need for RRT during hospital stay (RR: 1.13; 95% CI: 0.61, 2.09) (Figure S1A), following 30 days (RR: 1.78; 95% CI: 0.53, 6.01) (Figure S1B), 60 days (RR: 3.57; 95% CI: 0.75, 16.95) (Figure S1C), and 90 days (RR: 1.54; 95% CI: 0.06, 40.57) of hospital discharge (Figure S1D).

#### MAKE

Two studies reported the occurrence of MAKE. The application of care bundle did not significantly change the incidence of MAKE after 30 days (RR: 1.71; 95% CI: 0.94, 3.11) (Figure S2A), 60 days (RR: 1.57; 95% CI: 0.82, 3.03) (Figure S2B), and 90 days (RR: 1.19; 95% CI: 0.68, 2.10) (Figure S2C).

#### Mortality

Compared with standard care, the use of care bundle did not significantly change mortalities at hospital discharge (OR: 0.62; 95% CI: 0.15, 2.56) (Figure S3A), and after 30 days (OR: 0.82; 95% CI: 0.39, 1.73) (Figure S3B), and 60 days (RR: 1.22; 95% CI: 0.54, 2.77) (Figure S3C).

#### PRD

The use of care bundle significantly increased the risk of PRD compared with the controls after 30 days (RR: 2.39; 95% CI: 1.02; 5.58) (Figure S4).

#### Length of hospital and ICU stay

There were no significant differences between those under care bundle and standard care in terms of length of hospital stay (MD: -0.04; 95% CI: -0.18, 0.09) (Figure S5) and length of ICU stay (MD: 0.07; 95% CI: -0.09, 0.24) (Figure S6).

### Sensitivity analysis

The results of sensitivity analysis showed no significant difference between care bundle and standard care in terms of AKI occurrence (OR: 0.78; 95% CI: 0.55, 1.10) (Figure S7). However, bundle care significantly reduced the odds of severe AKI by 42% (OR: 0.58, 95% CI: 0.34, 0.98) (Figure S8).

### Quality assessment

The mean quality assessment score for the observational studies was 7.2. Out of five studies, two had an overall score of six [30, 31], one had seven [29], one had eight [28], and one had nine out of ten scores [32]. The sample size domain and reporting of non-respondents had the highest risk of bias (Table S3).

The overall bias for the two RCTs was some concerns. Both studies had a low risk of bias for all domains, except for the bias due to the deviation from the intended intervention [26, 27] (Table S4).

## Discussion

The study results revealed a significant reduction in both AKI and moderate-to-severe AKI following the implementation of care bundle measures. However, no notable changes were observed in the need for RRT, incidence of MAKE, length of hospital and ICU stays, or mortality among patients undergoing cardiac surgeries. Interestingly, the risk of PRD was higher in the care bundle group than in the standard care group. The results of the sensitivity analysis showed a significant reduced risk of severe AKI in those receiving care bundle.

Findings from a meta-analysis of eight studies indicated that the AKI care bundle non-significantly decreased the occurrence of AKI (RR: 0.90; 95% CI: 0.76, 1.05) [11]. For moderate-to-severe AKI, the same study reported a 22% reduction among those who received the care bundle [11]. Our results align with with the abovementioned study for moderate-to-severe AKI, and we also observed a 0.78-fold reduced incidence of AKI in care bundle recipients (95% CI: 0.61, 0.99). To explain this difference, it is important to note that the prior study included both RCTs and before-after studies on adult and pediatric populations across various setting, including ICUs, wards, and emergency departments [11]. A subgroup analysis of ICU patients in that study also show no significant association with AKI incidence (RR: 0.88; 95% CI: 0.71, 1.10) [11]. However, our analysis focused specifically on adult cardiac patients in ICUs. Nevertheless, the 95% CI for AKI occurrence in our study was close to the null value of one (95% CI: 0.61, 0.99). So, perhaps a future updated meta-analysis with higher number of primary studies can more clearly elucidate the association. Consistent with our findings, the systematic review by See and colleagues, which included diverse medical and surgical patients, demonstrated that the AKI care bundle significantly reduced moderate-to-severe AKI with an OR of 0.65 (95% CI: 0.51, 0.82). This association remained significant when the analysis was limited to RCTs only (OR: 0.55; 95% CI: 0.39, 0.79) [12].

In terms of PRD, not only did care bundle not reduce its occurrence, but also it increased the risk of PRD by 2.39 times. However, there were not significant differences between care bundle and standard care in terms of MAKE. The previous similar systematic reviews did not assess PRD as an outcome to compare our results [11, 12]. Maybe one of the reasons is that PRD is a rare condition following cardiac surgery, as the incidence of PRD after 60 and 90 days was zero in one of the included studies [27]. The definition of PRD or MAKE might also differ between studies which can explain the differences that despite a significant increase for PRD, MAKE did not show variations. Moreover, we only performed the meta-analysis on only two RCTs, which is not a satisfactory number of original studies to draw a conclusion. The compliance with care bundle was associated with improved outcomes for some other diseases. For instance, the number of ventilator-associated pneumonia was significantly decreased in those receiving ventilator care bundles compared with controls (OR: 0.42; 95% CI: 0.33, 0.54) [33]. Also, the application of care bundle decreased the incidence of central line-associated bloodstream infections by 60% (95% CI: 0.31, 0.51) [34]. However, it should be noted that the effects of care bundle on patient outcomes are still contradictory. Accordingly, a systematic review and meta-analysis showed no significant differences between care bundle and control measures in different types of negative patient outcomes (RR: 0.97; 95% CI: 0.71, 1.34) [35].

We did not find any significant association between the use of an AKI care bundle and the need for RRT, MAKE, and mortality at hospital discharge or in follow-ups. In this regard, Schaubroeck et al. revealed a non-significant decrease in the use of RRT among all patients (RR: 0.67; 95% CI: 0.38, 1.19) and patients hospitalized in ICUs (RR: 0.72; 95% CI: 0.41, 1.26) [11]. Furthermore, they did not find any significant association between the use of an AKI care bundle and in-hospital mortality (RR: 1.03; 95% CI: 0.73, 1.46) or mortality after 30 days (RR: 1.00; 95% CI: 0.42, 2.39) [11]. The other meta-analysis, which assessed the application of an AKI care bundle with or without using biomarkers, showed a non-significant reduction of all-cause mortality among those who used an AKI care bundle when it pooled the results of both RCTs and non-RCTs (OR: 0.88; 95% CI: 0.75, 1.02) or limited the findings to only RCTs (OR: 0.81; 95% CI: 0.44, 1.51) [12]. On the other hand, the occurrence of MAKEs significantly decreased by 27% (OR: 0.73; 95% CI: 0.66, 0.81) for all included studies and decreased by 45% (OR: 0.55; 95% CI: 0.41, 0.74) for only RCTs [12]. Variations in the inclusion criteria between the systematic reviews, in particular regarding the eligible population, might explain the reasons for the differences.

The length of hospital and ICU stays ranged did not show any significant differences between those who received care bundle and standard care. In this regard, the meta-analysis of three before-after studies showed no differences in the duration of hospital stay in those who used the AKI care bundle (MD: −0.65; 95% CI: -1.40, 0.09) [11]. Regarding the use of ventilatory bundle, a meta-analysis on seriously ill adult populations showed a significantly reduced duration of ICU stay by 2.57 days (*p* = 0.03) [36]. Similarly, results of an observational study on 22 patients with chronic obstructive pulmonary disease who received care bundle and 22 controls showed a reduced duration of hospital stay among cases than controls (51.2 vs. 101.1 h; *p* = 0.001) [37]. In contrast, there were no significant changes in the duration of hospital stay (MD: -0.37; 95% CI: -1.47, 0.74) and ICU stay (MD: 0.07; 95% CI: -0.40, 0.54) among those undergoing the ventilator care bundle in comparison with the standard care [33]. Generally, the contrasting results between the studies make it hard to draw an overall conclusion about the roles of care bundle, especially AKI care bundle, on the length of hospital and ICU stay. This highlights the importance of conducting future studies on this topic.

The compliance with the care bundle was only reported in three included studies which was between about 35% (for at least three components) and 65%. Another systematic review on 23 studies revealed a high range for compliance to care bundle from 8 to 100% [11]. Results of a scoping review showed higher number of components of bundle intervention and intricacy of each component were correlated with reduced compliance [38]. They suggested the adoption of evaluative and iterative methods, the establishment of stakeholder relationships, and the implementation of training approaches as measures to increase the compliance with the care bundle [38]. Among nursing teams, fatigue due to the implementation of care bundles led to the introduction of “bundle fatigue” which can be also another obstacle in the proper application of care bundles among nursing teams [39]. Comprehensive strategies, involving both organizational (e.g., multidisciplinary teams and enhancement of health recording systems) and system intervention (e.g., incentives and recognition) can be more effective in increasing the adherence to care bundles [40]. In ICU settings, training, reminders, and performance assessment and feedback were the most common applied approaches to improve the adherence to care bundle [41]. For the sepsis bundle, implementations of programs to improve performance increased care bundle compliance by more than 2–4 times [42]. As a result, these strategies might also be effective in AKI care bundles, warranting future investigations.

While the care bundle intervention appeared effective in reducing AKI incidence, publication bias may influence the observed impact on stage 2–3 AKI outcomes. Egger’s test revealed significant publication bias for stage 2–3 AKI (*p* = 0.03), suggesting that studies with favorable or statistically significant results may be disproportionately represented. This bias could result in an overestimation of the intervention’s effectiveness for more severe AKI stages, as studies with null or less significant findings may have been underreported or unpublished. In contrast, no publication bias was detected for all-stage AKI (*p* = 0.77), indicating more balanced reporting across studies regardless of outcome significance for overall AKI incidence. Acknowledging this potential bias is important, as it affects the reliability of conclusions specifically for stage 2–3 AKI. Future meta-analyses would benefit from a broader inclusion of studies across all outcome types, including those with non-significant findings, to better assess the true impact of care bundle interventions on more severe AKI stages.

The quality assessment indicated that biases within individual studies could have meaningful implications for the overall findings and recommendations of this review. The observational studies, which received a mean quality score of 7.2, displayed considerable variability in methodological rigor. Two studies scored only six out of ten, primarily due to high risk of bias in the sample size and the handling of non-respondent data. These limitations are significant because small sample sizes can reduce statistical power, potentially leading to less precise or even skewed effect estimates that may not accurately reflect true relationships. Additionally, inadequate reporting on non-respondents has a risk of selection bias if those who did not participate or complete the study differ in key characteristics (such as disease severity or intervention response) from those who did. Such biases could result in an over- or underestimation of observed effects, ultimately affecting the reliability of conclusions drawn from these studies. In contrast, both RCTs demonstrated a low risk of bias across most domains, but there was some concern related to deviations from intended interventions. This specific type of bias could dilute the effect sizes reported, as unintended variations in intervention delivery might reduce the observed impact of the treatments. In cases where deviations occurred, they could potentially lead to discrepancies between the intended intervention outcomes and the actual study results, complicating direct comparisons across studies. This concern is critical, as even minor deviations may impact the validity of outcome measures, influencing the pooled effect in meta-analyses or systematic reviews. Overall, these biases collectively reduce the strength of the recommendations that can be made from the findings. While the RCTs add robust support for certain interventions, the moderate-to-high risk of bias in several observational studies suggests that results should be interpreted with caution. For future studies, addressing sample size calculation, improving transparency around non-respondent data, and ensuring consistent intervention protocols will be crucial for enhancing the reliability of evidence and enabling more definitive clinical recommendations.

Overall, the findings indicate that there is scant, limited, and heterogeneous data on the application of AKI care bundle on kidney outcomes. Other interventions for AKI prevention, such as AKI nurses and AKI teams, electronic alerts, education, smart phone applications for AKI, and sick day rules have been suggested as interventions for the prevention from AKI [43]. Combinations of the interventions, especially electronic alerts and AKI bundles, complemented by educational support are recommended as one of the most effective approaches for AKI prevention [43]. Among the individual bundle components, sufficient systemic blood pressure and cardiac output are more important than other components like nephrotoxic drugs, as hypotension and cardiac index less than three significantly increase the risk of AKI by 2.37 and 1.97 times, respectively [44].

Cardiac surgery-associated AKI remains a significant complication, and various strategies have been explored to reduce its incidence and improve outcomes. Non-pharmacological approaches, such as remote ischemic preconditioning, are particularly promising. This approach involves brief, controlled episodes of ischemia that can increase the kidney’s resilience to stress, and recent meta-analyses indicated that it effectively reduced cardiac surgery-associated AKI incidence and ICU stays while lowering the rates of post-surgical dialysis requirements, though further studies are needed to confirm its long-term benefits [45]. Technologies like electronic alert systems have also proven valuable by improving clinical responses to AKI. For example, electronic alerts are associated with a 45% increase in nephrology consultations (RR, 1.45; 95% CI, 1.04–2.02) and a 25% reduction in post-AKI non-steroidal anti-inflammatory drug use (RR, 0.75; 95% CI, 0.59–0.95), thereby enhancing proactive management of AKI risk factors [46]. Although electronic alerts have not been associated with a significant reduction in mortality (RR, 0.96; 95% CI, 0.89–1.03), they still play a role in mitigating AKI progression, as demonstrated by a modest 9% reduction in AKI stage progression (RR, 0.91; 95% CI, 0.84–0.99) [46]. Pharmacologically, sodium-glucose cotransporter 2 (SGLT2) inhibitors, particularly empagliflozin, have shown potential for renal protection by targeting inflammation, mitochondrial dysfunction, and oxidative stress—key factors in cardiac surgery-associated AKI. A trial is currently investigating empagliflozin’s specific efficacy in reducing adverse kidney outcomes after cardiac surgery, which could mark a new avenue for AKI prevention if confirmed effective [47]. A network meta-analysis determined several strategies have been found effective in preventing post-cardiac surgery AKI. Natriuretic peptides were the most effective pharmacological treatment, reducing AKI incidence by 70% and lowering mortality by 50%. Other pharmacological treatments like fenoldopam, erythropoietin, and levosimendan also reduced AKI risk and the need for dialysis. Among non-pharmacological interventions, remote ischemic preconditioning lowered AKI incidence by 24% [48]. Together, these diverse methods highlight the potential of a multi-modal strategy for cardiac surgery-associated AKI prevention, combining non-pharmacological, technological, and pharmacological interventions.

To our best of knowledge, this study appears to be the first systematic review and meta-analysis which focused on patients with cardiac diseases in order to evaluate effects of care bundle on kidney-related outcomes. We used a comprehensive search strategy across multiple databases, including grey literature, which strengthens the robustness of the literature review. Furthermore, adherence to PRISMA guidelines for systematic reviews ensures the transparency and reproducibility of the methodology. The findings can be helpful for health policymakers and nursing teams to provide evidence-based and high quality cares in ICUs. Nevertheless, we acknowledge that the study has several limitations that should be taken into account in the interpretation of the study findings. First, there were a limited number of studies with small sample sizes. We only included five studies in meta-analysis. For MAKE, PRD, RRT, and mortality outcomes, there were only two eligible studies. Given the limited number of studies and small sample sizes in the meta-analysis, the findings should be interpreted with caution, as the lack of a sufficient number of events and participants may undermine the robustness and generalizability of the conclusions. This might limit the statistical power and the robustness of conclusions drawn. Moreover, the evaluation of publication bias for those outcomes was not applicable. The limited number of studies also prevented us from performing subgroup analysis. A limitation of this meta-analysis is the potential for type I error due to heterogeneity among included studies. Second, we could not gain access to the full text of one study, despite contacting the corresponding author. Third, most of the studies were carried out in Europe, and it should be considered in the generalizability of the findings to other continents. Next observational or clinical trials in other countries in Asia or America are suggested. Fourth, the included studies exhibited significant heterogeneity in terms of design (e.g., RCTs vs. observational studies), care bundle definitions, and outcomes measured. This variation complicated the synthesis of results and limits the generalizability of our findings across different clinical settings. Future research should aim to standardize care bundle definitions and outcome measures to reduce heterogeneity and improve the reliability of results in similar populations. Fifth, another limitation is the lack of consistent reporting on compliance with care bundles across the included studies. Only a few studies reported adherence to care bundle protocols, raising concerns about the reliability of the interventions being evaluated. Future updated systematic reviews are suggested to consider the point. Sixth, another limitation was the variability in how AKI was defined across the included studies, with most of them using the KDIGO criteria and only two applied others other definitions. This inconsistency in AKI definitions may have introduced variability in how outcomes were reported and may affect the comparability of results across studies. It is important for future research to standardize the definition of AKI in order to improve the comparability of results and enhance the generalizability of findings. Moreover, there could be the lack of standardized outcome reporting related to AKI. Uniformity in outcome definitions and reporting would facilitate improved comparisons and enable more robust meta-analyses, which are essential for synthesizing evidence across studies. This point should be considered in designing future primary studies. Seventh, our analysis provides limited exploration of the mechanisms through which care bundles might influence outcomes. Future research would benefit from exploring these mechanisms in more detail, which could help clarify the pathways through which care bundles impact patient outcomes. Eighth, most of the included studies focused on short-term outcomes (e.g., 30 to 90 days), and long-term follow-up data on AKI and the effects of care bundles are lacking. Future studies with long-term follow-up are needed to fully understand the lasting implications of care bundles on kidney-related outcomes. In addition, another limitation of this study is the combination of distinct care bundles—KDIGO and ERACS—under a single AKI care bundle category, despite their differing focuses; this may introduce variability, as each protocol targets unique aspects of postoperative management and kidney protection. Furthermore, one of the included studies (i.e., the study by Khoury et al. [28]) examined PCI patients, who may have a different risk profile compared to those undergoing cardiac surgery. While both groups share common risk factors for AKI, such as nephrotoxic exposure and hemodynamic challenges, their procedural differences could introduce heterogeneity into the findings. This inclusion was intended to enhance the generalizability of our findings across various high-risk cardiac populations, but it should be considered when interpreting the results.

## Conclusions

Among critically ill patients, including those undergoing cardiac surgeries, the application of care bundles appears to be effective in reducing the incidence of AKI, particularly in moderate to severe cases. However, as some studies included non-cardiac critical illness populations, the observed benefit may not be exclusively due to effects within cardiac surgery patients. Given the limited number of studies and small sample sizes, caution is advised in generalizing these findings broadly to clinical practice. An in-depth analysis of compliance with care bundles should be considered in future studies. Additionally, qualitative studies that explore the experiences of healthcare providers and patients with AKI care bundles could provide valuable insights into barriers and facilitators of implementation, which can be considered in next studies to inform future strategies for broader adoption. Future large-scale RCTs focusing specifically on cardiac surgery patients are needed, along with updated meta-analyses, to more precisely evaluate the impact of care bundles on AKI in this population.

## Electronic supplementary material

Below is the link to the electronic supplementary material.

Supplementary Material 1

Supplementary Material 2

## Data Availability

The data that supports the findings of this study are available in the supplementary material of this article.
