# Supplementary material for: Effect of acute kidney injury care bundle on kidney outcomes in cardiac patients receiving critical care: a systematic review and meta-analysis
Source: BMC Nephrol. 2025 Jan 10;26:17. doi: 10.1186/s12882-025-03955-1 (PMC11721091; doi:10.1186/s12882-025-03955-1)
Supplement: Supplementary file 1 — Supplementary Material 1 [file 12882_2025_3955_MOESM1_ESM.docx]

**Table S1.** Search strategies for PubMed, Scopus, Web of Science, EMBASE, Google scholar, and clinicaltrials.gov website.

| **Step** | **Search strategy** | **Results (Search date: Nov 9, 2024)** |
| --- | --- | --- |
| **PubMed** | | |
| 1 | "Cardiovascular Diseases"[Mesh] OR "Heart Diseases"[Mesh] OR "Myocardial Ischemia"[Mesh] OR "Myocardial Infarction"[Mesh] OR "Cardiovascular Disease"[Title/Abstract] OR "Cardiac disease"[Title/Abstract] OR "cardiac event"[Title/Abstract] OR "heart disease"[Title/Abstract] OR "Myocardial Ischemia"[Title/Abstract] OR "Ischemic Heart Disease"[Title/Abstract] OR "coronary disease"[Title/Abstract] OR "Myocardial Infarction"[Title/Abstract] OR "heart attack"[Title/Abstract] OR "Myocardial Infarct"[Title/Abstract] OR "Cardiovascular Stroke"[Title/Abstract] OR Cardiac[Title/Abstract] OR Heart[Title/Abstract] | 3,619,140 |
| 2 | "Patient Care Bundles"[Mesh] OR "Evidence-Based Practice"[Mesh] OR "Care bundle"[Title/Abstract] OR "Patient Care Bundle"[Title/Abstract] OR "Bundle"[Title/Abstract] OR "Clinical bundle"[Title/Abstract] OR "Protocol bundle"[Title/Abstract] OR "Intervention bundle"[Title/Abstract] OR "Healthcare bundle"[Title/Abstract] OR "Evidence-based care"[Title/Abstract] OR "Clinical practice guideline"[Title/Abstract] OR "Care checklist"[Title/Abstract] OR "Evidence-Based Healthcare"[Title/Abstract] OR "Evidence Based Healthcare"[Title/Abstract] OR "Evidence-Based Practice"[Title/Abstract] OR "Evidence Based Practice"[Title/Abstract] | 162,247 |
| 3 | "Acute Kidney Injury"[Mesh] OR "Renal Dialysis"[Mesh] OR "Renal Replacement Therapy"[Mesh] OR "kidney injur*"[Title/Abstract] OR "Acute Kidney Injury"[Title/Abstract] OR "Acute Renal Injury"[Title/Abstract] OR "Acute Kidney Injuries"[Title/Abstract] OR "Acute renal Injuries"[Title/Abstract] OR "Acute Renal Insufficiency"[Title/Abstract] OR "Acute Renal Insufficiencies"[Title/Abstract] OR "Acute kidney Insufficiency"[Title/Abstract] OR "Acute kidney Insufficiencies"[Title/Abstract] OR "Acute Renal Failure"[Title/Abstract] OR "Acute kidney Failure"[Title/Abstract] OR AKI[Title/Abstract] OR ARF[Title/Abstract] OR "RIFLE criteria"[Title/Abstract] OR "KDIGO guidelines"[Title/Abstract] OR "Renal Dialysis"[Title/Abstract] OR "Renal Dialyses"[Title/Abstract] OR Hemodialysis[Title/Abstract] OR Hemodialyses[Title/Abstract] OR Haemodialysis[Title/Abstract] OR Dialysis[Title/Abstract] OR Dialyses[Title/Abstract] OR "Renal Replacement"[Title/Abstract] OR "Kidney Replacement"[Title/Abstract] | 403,778 |
| 4 | #1 AND #2 AND #3 | 438 |
| **Scopus** | | |
| 1 | TITLE-ABS-KEY("Cardiovascular Disease" OR "Heart Disease" OR "Myocardial Ischemia" OR "Myocardial Infarction" OR "Cardiac disease" OR "cardiac event" OR "Ischemic Heart Disease" OR "coronary disease" OR "heart attack" OR "Myocardial Infarct" OR "Cardiovascular Stroke" OR Cardiac OR Heart) | 3,400,908 |
| 2 | TITLE-ABS-KEY("Patient Care Bundle" OR "Evidence-Based Practice" OR "Care bundle" OR "Bundle" OR "Clinical bundle" OR "Protocol bundle" OR "Intervention bundle" OR "Healthcare bundle" OR "Evidence-based care" OR "Clinical practice guideline" OR "Care checklist" OR "Evidence-Based Healthcare" OR "Evidence Based Healthcare" OR "Evidence Based Practice") | 334,817 |
| 3 | TITLE-ABS-KEY("Acute Kidney Injury" OR "Renal Dialysis" OR "Renal Replacement Therapy" OR "kidney injur*" OR "Acute Renal Injury" OR "Acute Kidney Injuries" OR "Acute renal Injuries" OR "Acute Renal Insufficiency" OR "Acute Renal Insufficiencies" OR "Acute kidney Insufficiency" OR "Acute kidney Insufficiencies" OR "Acute Renal Failure" OR "Acute kidney Failure" OR AKI OR ARF OR "RIFLE criteria" OR "KDIGO guidelines" OR "Renal Dialyses" OR Hemodialysis OR Hemodialyses OR Haemodialysis OR Dialysis OR Dialyses OR "Renal Replacement" OR "Kidney Replacement") | 483,267 |
| 4 | #1 AND #2 AND #3 | 1,436 |
| **Web of Science** | | |
| 1 | TS=("Cardiovascular Disease" OR "Heart Disease" OR "Myocardial Ischemia" OR "Myocardial Infarction" OR "Cardiac disease" OR "cardiac event" OR "Ischemic Heart Disease" OR "coronary disease" OR "heart attack" OR "Myocardial Infarct" OR "Cardiovascular Stroke" OR Cardiac OR Heart) | 2,341,428 |
| 2 | TS=("Patient Care Bundle" OR "Evidence-Based Practice" OR "Care bundle" OR "Bundle" OR "Clinical bundle" OR "Protocol bundle" OR "Intervention bundle" OR "Healthcare bundle" OR "Evidence-based care" OR "Clinical practice guideline" OR "Care checklist" OR "Evidence-Based Healthcare" OR "Evidence Based Healthcare" OR "Evidence Based Practice") | 136,386 |
| 3 | TS=("Acute Kidney Injury" OR "Renal Dialysis" OR "Renal Replacement Therapy" OR "kidney injur*" OR "Acute Renal Injury" OR "Acute Kidney Injuries" OR "Acute renal Injuries" OR "Acute Renal Insufficiency" OR "Acute Renal Insufficiencies" OR "Acute kidney Insufficiency" OR "Acute kidney Insufficiencies" OR "Acute Renal Failure" OR "Acute kidney Failure" OR AKI OR ARF OR "RIFLE criteria" OR "KDIGO guidelines" OR "Renal Dialyses" OR Hemodialysis OR Hemodialyses OR Haemodialysis OR Dialysis OR Dialyses OR "Renal Replacement" OR "Kidney Replacement") | 351,630 |
| 4 | #1 AND #2 AND #3 | 278 |
| **Embase** | | |
| 1 | 'Heart Disease'/exp OR 'heart muscle ischemia'/exp OR 'heart infarction'/exp OR 'Cardiac disease':ti,ab,kw OR 'cardiac event':ti,ab,kw OR 'heart disease':ti,ab,kw OR 'Myocardial Ischemia':ti,ab,kw OR 'Ischemic Heart Disease':ti,ab,kw OR 'Myocardial Infarction':ti,ab,kw OR 'heart attack':ti,ab,kw OR 'Myocardial Infarct':ti,ab,kw OR 'Cardiac':ti,ab,kw OR 'Heart':ti,ab,kw | 3,577,494 |
| 2 | 'care bundle'/exp OR 'Care bundle':ti,ab,kw OR 'Patient Care Bundle':ti,ab,kw OR 'Bundle':ti,ab,kw OR 'Clinical bundle':ti,ab,kw OR 'Protocol bundle':ti,ab,kw OR 'Intervention bundle':ti,ab,kw OR 'Healthcare bundle':ti,ab,kw OR 'Evidence-based care':ti,ab,kw OR 'Clinical practice guideline':ti,ab,kw OR 'Care checklist':ti,ab,kw OR 'Evidence-Based Healthcare':ti,ab,kw OR 'Evidence Based Healthcare':ti,ab,kw OR 'Evidence-Based Practice':ti,ab,kw OR 'Evidence Based Practice':ti,ab,kw | 106,262 |
| 3 | 'acute kidney failure'/exp OR 'hemodialysis'/exp OR 'renal replacement therapy'/exp OR 'kidney injur*':ti,ab,kw OR 'Acute Kidney Injury':ti,ab,kw OR 'Acute Renal Injury':ti,ab,kw OR 'Acute Kidney Injuries':ti,ab,kw OR 'Acute renal Injuries':ti,ab,kw OR 'Acute Renal Insufficiency':ti,ab,kw OR 'Acute Renal Insufficiencies':ti,ab,kw OR 'Acute kidney Insufficiency':ti,ab,kw OR 'Acute kidney Insufficiencies':ti,ab,kw OR 'Acute Renal Failure':ti,ab,kw OR 'Acute kidney Failure':ti,ab,kw OR 'AKI':ti,ab,kw OR 'ARF':ti,ab,kw OR 'RIFLE criteria':ti,ab,kw OR 'KDIGO guidelines':ti,ab,kw OR 'Renal Dialysis':ti,ab,kw OR 'Renal Dialyses':ti,ab,kw OR 'Hemodialysis':ti,ab,kw OR 'Hemodialyses':ti,ab,kw OR 'Haemodialysis':ti,ab,kw OR 'Dialysis':ti,ab,kw OR 'Dialyses':ti,ab,kw OR 'Renal Replacement':ti,ab,kw OR 'Kidney Replacement':ti,ab,kw | 526,032 |
| 4 | #1 AND #2 AND #3 | 555 |
| **Google Scholar (Search date: Nov 9, 2024)** | | |
| 1 | (“Cardiac" OR Heart) AND (“Bundle Care” OR "care bundle") AND ("kidney injury" OR "renal injury" OR hemodialysis OR "replacement therapy" OR AKI OR ARF) | About 4,510 |
| **Clinicaltrials.gov (Search date: Jan 4, 2024)** | | |
| 1 | Condition: Cardiovascular Disease, Other terms: Bundle Care, Intervention/treatment: Care Bundle | 40 |

**Table S2.** Definition of KDIGO, ERACS, and AKI-CB care bundle.

| **Care bindle protocol** | **Bundle element** | **Descriptions** |
| --- | --- | --- |
| KDIGO | Discontinuation of all nephrotoxic agents | Not receive any nephrotoxic substances |
|  | Optimization of hemodynamics | Lowest documented mean arterial pressure >65mmHg and no treatment initiation or optimization |
|  | Close monitoring of serum creatinine, urine output and fluid balance | Serum creatinine measuring twice daily  Urine output recording at least every 2 hours  Fluid balance documenting twice daily |
|  | Avoidance of hyperglycemia | Blood glucose not ≥150mg/dl on two consecutive samples >3 hours |
|  | Consideration of alternatives to radiocontrast agents | Not receiving radiocontrast agents for the first 72 hours post-surgery |
|  | Discontinuation of angiotensin-converting-enzyme inhibitors and Angiotensin II Receptor Blockers | Not receiving ACEi/ARBs during the first 48 hours post-surgery |
|  | Avoidance of hydroxyethyl starch (HES), gelatin, and chloride-rich solutions | Not receiving HES, gelatine or chloride rich solutions for 72 hours post-surgery |
| ERACS (Enhanced Recovery After Cardiac Surgery) | \| Preoperative period: Patient Education and Lifestyle Changes \| \| --- \| | \| Provide detailed counseling, smoking and alcohol cessation, physical/mental prehabilitation (exercises, diet, psychotherapy) before surgery. \| \| --- \| |
|  | \| Management of Anemia \| \| --- \| \|  \| | \| Screen for anemia and avoid elective surgery in patients with untreated anemia to minimize complications and mortality risks. \| \| --- \| |
|  | \| Medication Adjustment \| \| --- \| | \| Avoid benzodiazepines, use drugs that reduce opioid requirements (e.g., premedicate with pregabalin 75 mg or melatonin as needed). \| \| --- \| |
|  | \| Carbohydrate-Loading \| \| --- \| | \| Administer simple and complex carbohydrate fluids until the evening before surgery and up to 2 hours prior to OR entry to prevent hypovolemia, insulin resistance, and protein catabolism. \| \| --- \| |
|  | \| Intraoperative Period: Temperature Management \| \| --- \| | \| Maintain normothermia during surgery to avoid hypothermia-related complications (e.g., prolonged drug metabolism, coagulopathy, shivering, cognitive disturbances, infections). \| \| --- \| |
|  | \| Anesthetic Pharmacotherapy \| \| --- \| | \| Use inhalation anesthetics (e.g., sevoflurane, desflurane at 0.8–1.0 MAC) and/or propofol (3 mg/kg/h), with specific opioid options (sufentanil, fentanyl, or remifentanil). \| \| --- \| |
|  | \| Preemptive Analgesia \| \| --- \| | \| Administer intravenous non-opioid analgesics (e.g., metamizole 1g before skin incision) and ketamine (30 mg post-induction) to reduce opioid use and manage postoperative pain. \| \| --- \| |
|  | \| Local Anesthetic Infiltration \| \| --- \| | \| Inject 60 ml of levobupivacaine (30 ml on both sides of sternotomy, 15 ml around drains) to lower postoperative opioid requirements. \| \| --- \| |
|  | \| Protective Lung Ventilation \| \| --- \| | \| Implement low oxygen concentrations with tidal volume at 8 ml/kg of ideal body weight (calculated as height in cm - 100) and maintain PEEP at 5 cm H2O; apply lung recruitment maneuvers as needed to prevent respiratory failure and atelectasis. \| \| --- \| |
|  | \| Goal-Directed Fluid Therapy \| \| --- \| | \| Maintain balanced crystalloid solutions (1–3 ml/kg cc/h), avoid NaCl 0.9% to prevent hyperchloremic acidosis, and limit synthetic colloids (20 ml/kg/day). Monitor blood chlorine and avoid excessive transfusions; allow intraoperative permissive oliguria. \| \| --- \| |
|  | \| Blood Glucose Management \| \| --- \| | \| Keep glucose levels between 140–180 mg/dl, with more restrictive control in select patients to minimize infection and cardiac risks. \| \| --- \| |
|  | \| Postoperative Period: Early Oral Liquid Intake \| \| --- \| | \| Initiate oral fluids as soon as possible, with a target of 1.5–1.7 L/day to prevent complications and shorten hospital stay. \| \| --- \| |
|  | \| Postoperative Feeding \| \| --- \| | \| Start oral feeding within 24 hours post-surgery to reduce insulin resistance, nitrogen excretion, muscle mass loss, and infection risks. \| \| --- \| |
|  | \| Prevention and Management of PONV \| \| --- \| | \| Assess risk using Apfel score, use at least two antiemetics (dexamethasone and ondansetron) if necessary, and avoid routine use of gastric tubes. \| \| --- \| |
|  | \| Multimodal Analgesia \| \| --- \| | \| Provide tailored, multimodal pain management with the eCASH (Comfort, Cooperation, Calm) approach; early analgesia, minimal sedation, and humane care. \| \| --- \| |
|  | \| Delirium Prevention \| \| --- \| | \| Administer preventive doses of haloperidol or atypical neuroleptics (e.g., risperidone, quetiapine) in high-risk patients, manage triggering factors like pain and hypotension. \| \| --- \| |
|  | \| Prevention of Atrial Fibrillation \| \| --- \| | \| Administer β-blockers perioperatively, individualizing timing and dosage to prevent adverse events. \| \| --- \| |
|  | \| Early Goal-Directed Mobilization \| \| --- \| | \| Initiate physical exercise and walking on the day of surgery; for cardiac surgery patients, start active mobilization on the first postoperative day. \| \| --- \| |
| AKI-CB (Acute Kidney Injury Care Bundle) | \| **Daily sCr Monitoring** \| \| --- \| | \| Monitor serum creatinine (sCr) at a fixed time daily (07:00 am) to detect early AKI changes. \| \| --- \| |
|  | \| **Electronic Reporting of sCr Changes** \| \| --- \| | \| Clinicians must document any sCr change in the electronic medical record, emphasizing even small variations from baseline to identify potential subclinical AKI. \| \| --- \| |
|  | \| **Baseline Comparison and Delta sCr Calculation** \| \| --- \| | \| Compare daily sCr to baseline, calculating delta sCr to track deviations from baseline values. Identify and flag minor sCr changes that could signal early AKI. \| \| --- \| |
|  | \| **Enhanced Monitoring in Response to AKI Detection** \| \| --- \| | \| If AKI is detected (sCr ≥ 0.3 mg/dl above baseline), increase sCr monitoring frequency to three times daily (07:00, 15:00, and 23:00) to closely follow kidney function trends. \| \| --- \| |
|  | \| Holding Nephrotoxic Medications \| \| --- \| | \| Discontinue nephrotoxic medications until sCr levels return closer to baseline to mitigate further kidney damage. \| \| --- \| |
|  | \| Early Nephrologist Consultation \| \| --- \| | \| Arrange a nephrologist consult if elevated sCr persists (> 0.3 mg/dl above baseline) over two consecutive measurements, to initiate specialized care as needed. \| \| --- \| |

**Table S3.** Risk of bias assessment for the included cross-sectional studies.

| Study ID | 1.Representativeness of the exposed cohort  a) Truly representative of the average in the target population. * (all subjects or  random sampling)  b) Somewhat representative of the average in the target population. * (nonrandom sampling)  c) Selected group of users.  d) No description of the sampling strategy. | 2. Sample size:  a) Justified and satisfactory. *  b) Not justified. | 3 Non-respondents:  a) Comparability between respondents and non-respondents characteristics is  established, and the response rate is satisfactory. *  b) The response rate is unsatisfactory, or the comparability between respondents  and non-respondents is unsatisfactory.  c) No description of the response rate or the characteristics of the responders and  the non-responders. | 4. Ascertainment of the exposure (risk factor):  a) Validated measurement tool. **  b) Non-validated measurement tool, but the tool is available or described.*  c) No description of the measurement tool. | 5. Comparability: (Maximum 2 stars)  1) The subjects in different outcome groups are comparable, based on the study design  or analysis. Confounding factors are controlled.  a) The study controls for the most important factor (select one). *  b) The study control for any additional factor. * | 6.Assessment of outcome  a) Independent blind assessment. **  b) Record linkage. **  c) Self report. *  d) No description. | 7) Statistical test:  a) The statistical test used to analyze the data is clearly described and  appropriate, and the measurement of the association is presented, including  confidence intervals and the probability level (p value). *  b) The statistical test is not appropriate, not described or incomplete. | Overall  Score |
| --- | --- | --- | --- | --- | --- | --- | --- | --- |
| Fleming et al. 2016 (1) | B | A | C | A | A, B | A | A | 9 |
| Engelman et al. 2020 (2) | B | B | C | B | A | B | A | **6** |
| Hoogma et al. 2022 (3) | B | B | C | A | - | B | A | **6** |
| Khoury et al. 2023 (4) | B | B | C | A | A, B | B | A | **8** |
| Massoth et al. 2023 (5) | B | A | C | A | - | B | A | **7** |

**Selection (maximum five stars)**

1) Representativeness of the sample:

a) Truly representative of the average in the target population* (all subjects or random sampling)

b) Somewhat representative of the average in the target population* (non-random sampling)

c) Selected group of users

d) No description of the sampling strategy

2) Sample size:

a) Justified and satisfactory*

b) Not justified

3) Non-respondents:

a) Comparability between respondents and non-respondents characteristics is established, and the response rate is satisfactory*

b) The response rate is unsatisfactory, or the comparability between respondents and non-respondents is unsatisfactory

c) No description of the response rate or the characteristics of the responders and the non-responders

4) Ascertainment of the exposure (risk factor):

a) Validated measurement tool**

b) Non-validated measurement tool, but the tool is available or described*

c) No description of the measurement tool

**Comparability (maximum two stars)**

1) The subjects in different outcome groups are comparable, based on the study design or analysis. Confounding factors are controlled.

a) The study controls for the most important factor (select one). *

b) The study control for any additional factor. *

**Outcome (maximum three stars)**

1) Assessment of the outcome:

a) Independent blind assessment**

b) Record linkage**

c) Self report*

d) No description

2) Statistical test:

a) The statistical test used to analyze the data is clearly described and appropriate, and the measurement of the association is presented, including confidence intervals and the probability level (p value)*

b) The statistical test is not appropriate, not described or incomplete

**Table S4.** Risk of bias assessment for the included randomized controlled trials.

| Study ID | D1. Bias arising  from the  randomization  process | D2. Bias due to  deviations from  intended  interventions | D3. Bias due to  missing  outcome data | D4. Bias in  measurement  of the outcome | D5. Bias in  selection of the  reported result | Overall bias |
| --- | --- | --- | --- | --- | --- | --- |
| Meersch et al. 2017 (6) | Low risk | Some concerns | Low risk | Low risk | Low risk | Some concerns |
| Zarbock et al. 2021 (7) | Low risk | Some concerns | Low risk | Low risk | Low risk | Some concerns |

RoB2 overall risk of bias judgment

Low risk of bias → The study is judged to be at low risk of bias for all domains for this result.

Some concerns → The study is judged to raise some concerns in at least one domain for this result, but not to be at high risk of bias for any domain.

High risk of bias → The study is judged to be at high risk of bias in at least one domain, or to have some concerns for multiple domains in a way that substantially lowers confidence in the result.


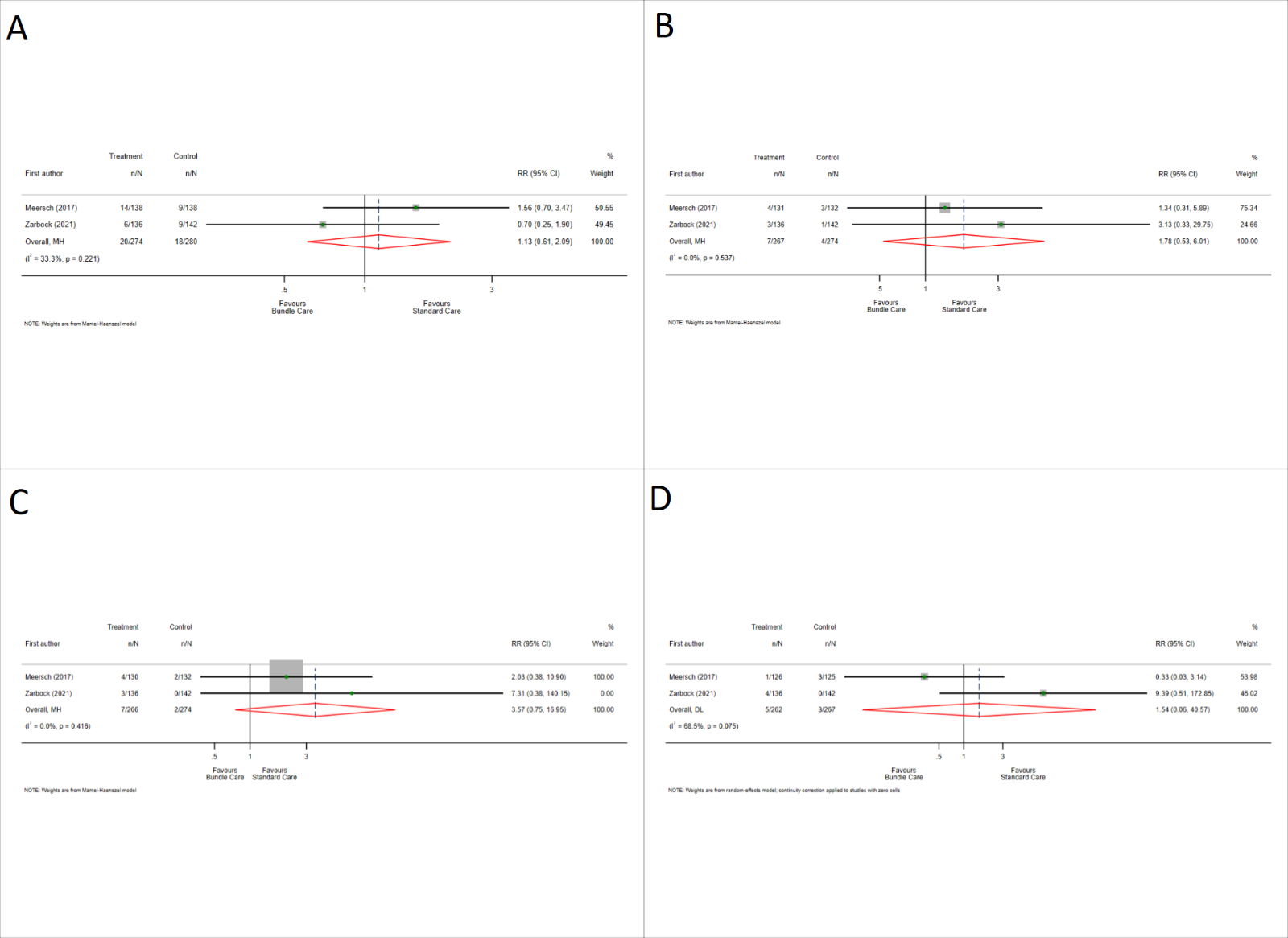


**Figure S1.** Forest plot of the association between receiving care bundle compared to standard care and the outcome of renal replacement therapy during hospital stay (A), following 30 days (B), 60 days (C), and 90 days (D) in participants with cardiac diseases. RR: risk ratio; CI: confidence interval.

**
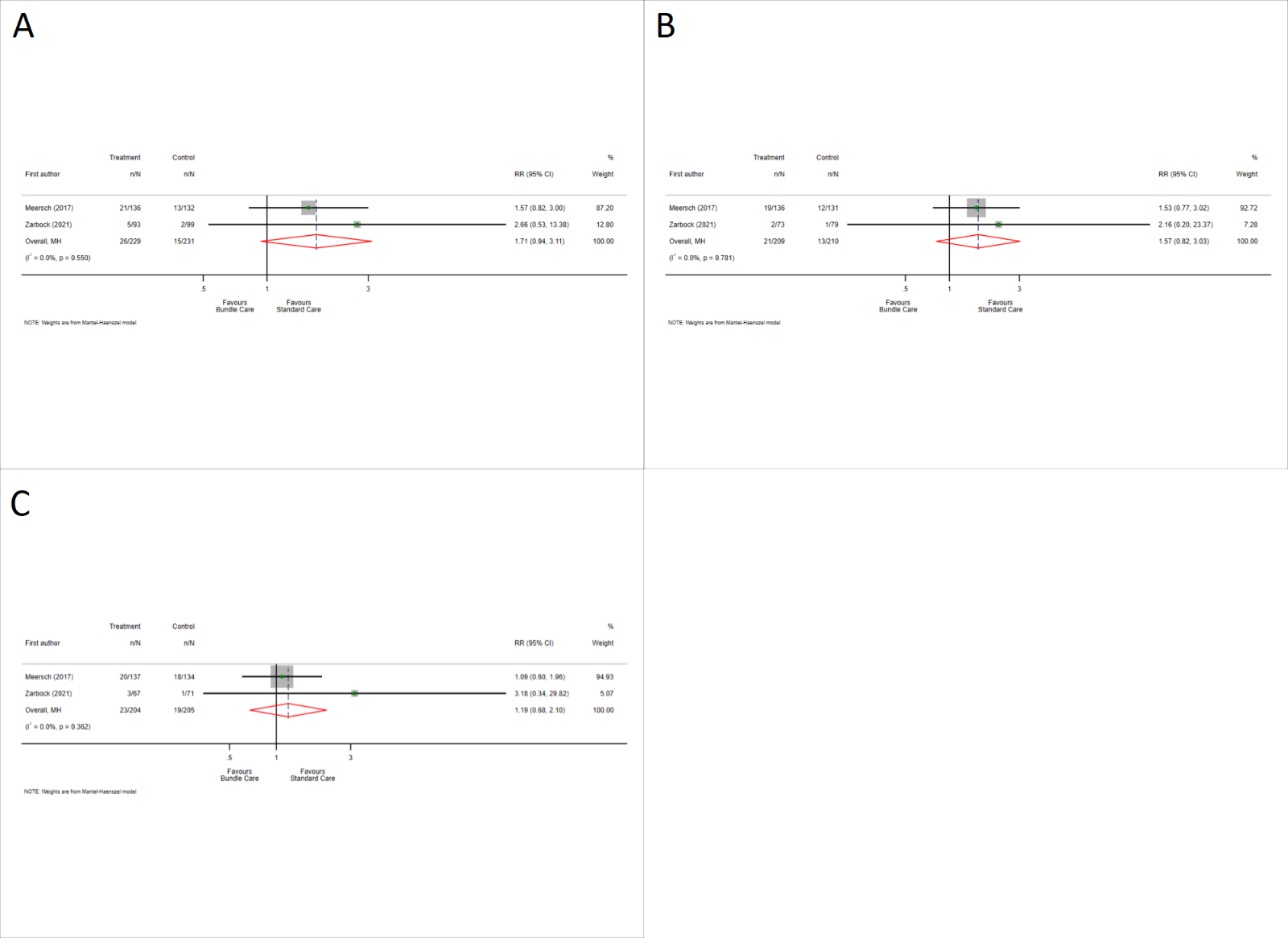
**

**Figure S2.** Forest plot of the association between receiving care bundle compared to standard care and the outcome of major adverse kidney events following 30 days (A), 60 days (B), and 90 days (C) in participants with cardiac diseases. RR: risk ratio; CI: confidence interval.


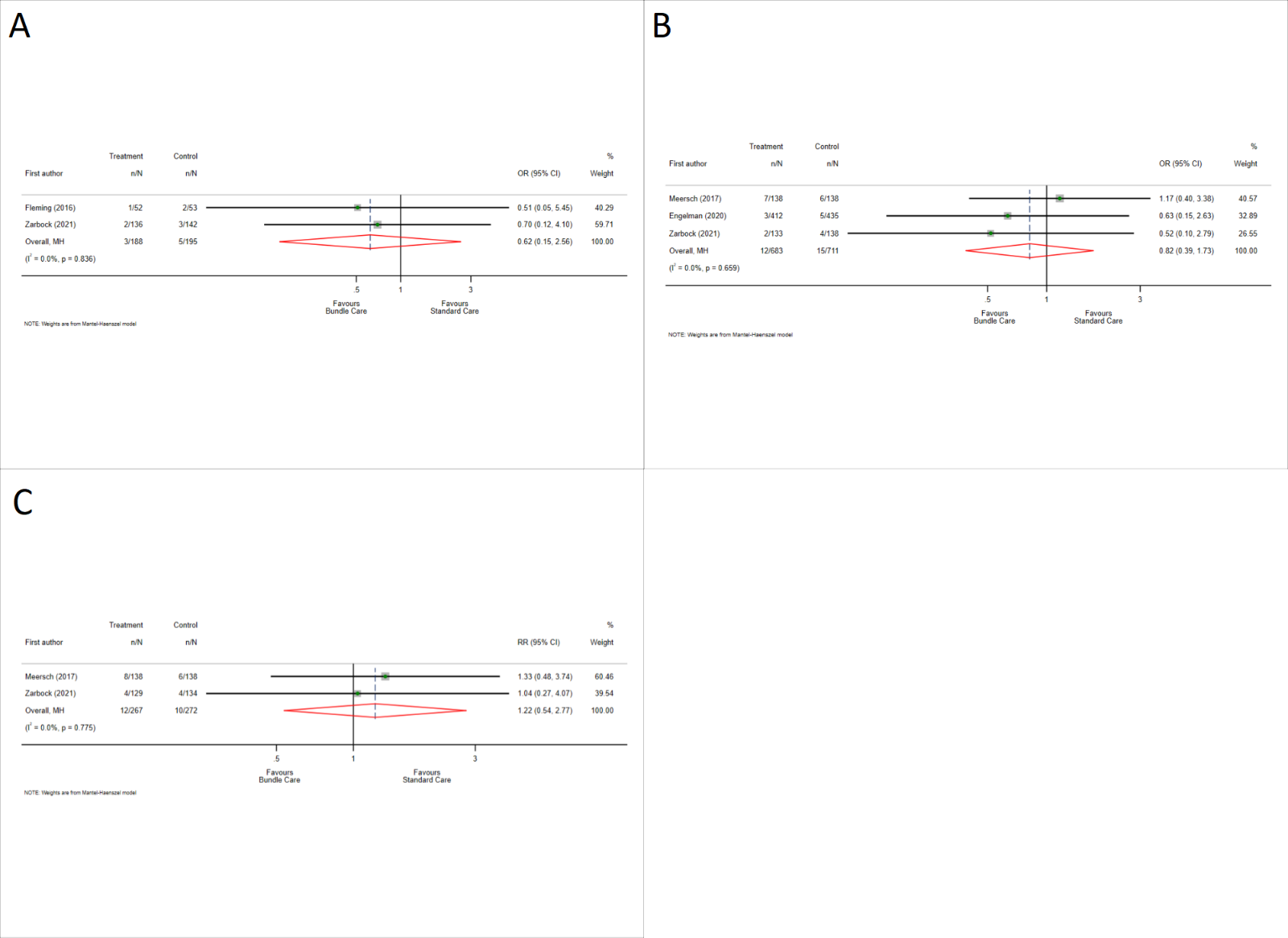


**Figure S3.** Forest plot of the association between receiving care bundle compared to standard care and the outcome of death at hospital discharge (A), and following 30 days (B), and 60 days (C) in participants with cardiac diseases. OR: odds ratio; RR: risk ratio; CI: confidence interval.


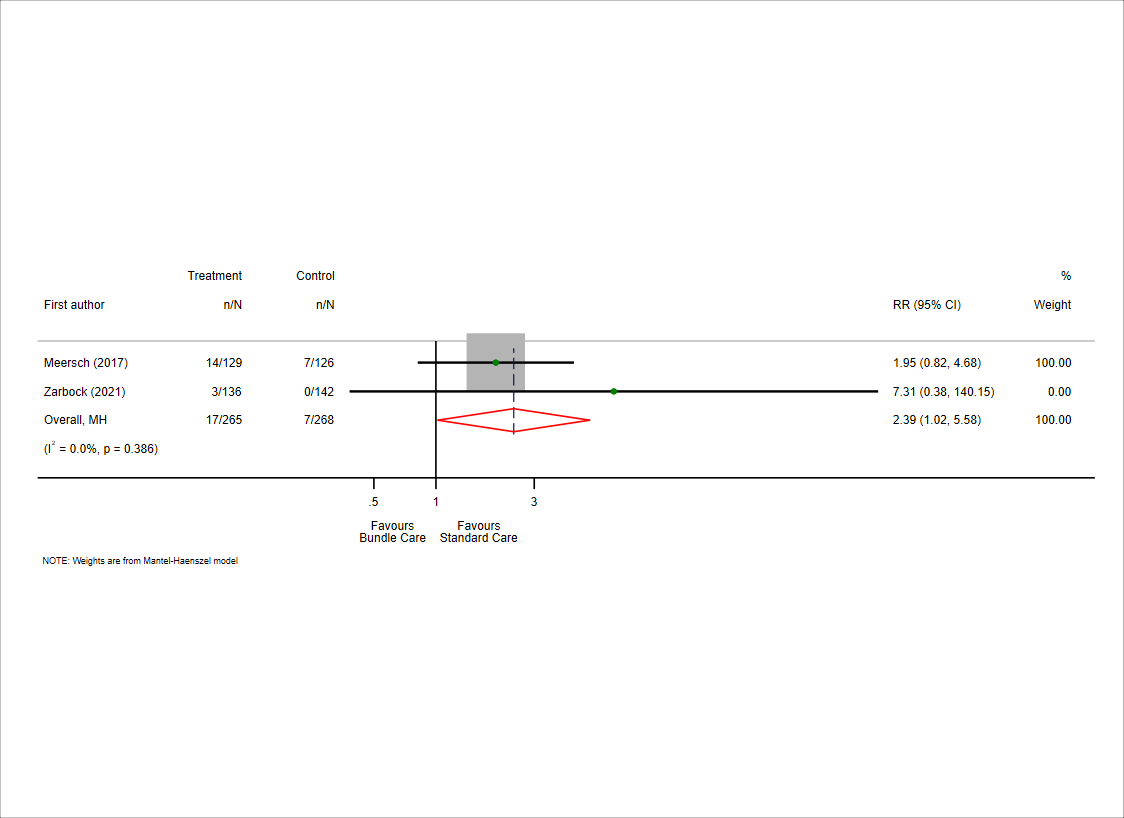


**Figure S4.** Forest plot of the association between receiving care bundle compared to standard care and the outcome of persistent renal dysfunction following 30 days in participants with cardiac diseases. RR: risk ratio; CI: confidence interval.


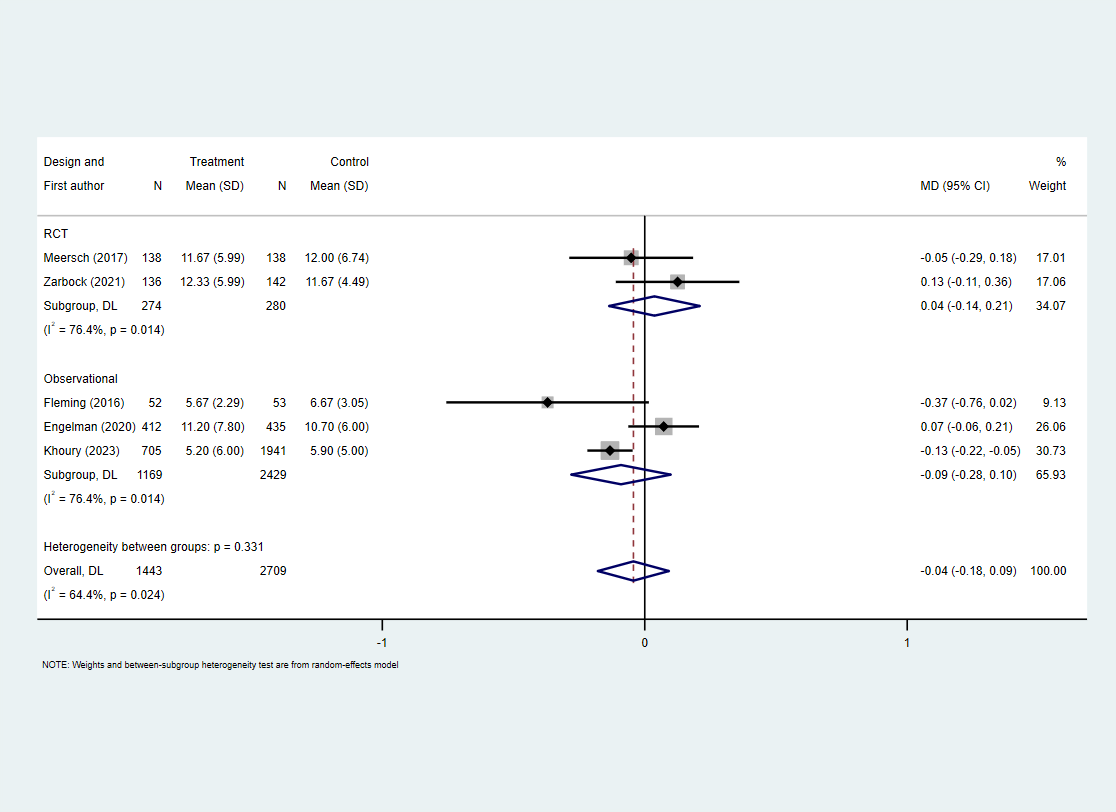


**Figure S5.** Forest plots of the association between receiving care bundle compared to standard care and the length of hospital stay. MD: mean difference; CI: confidence interval; DL: DerSimonian and Laird.


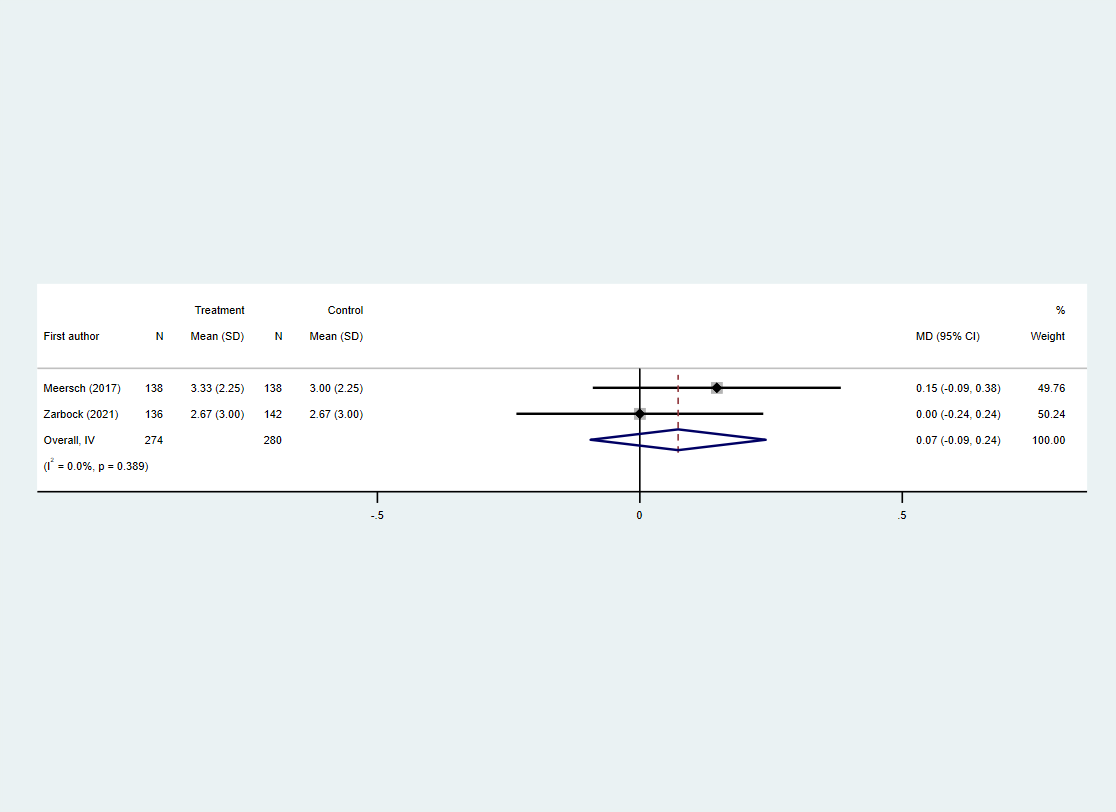


**Figure S6.** Forest plots of the association between receiving care bundle compared to standard care and the length of intensive care unit stay. MD: mean difference; CI: confidence interval; IV: inverse variance.


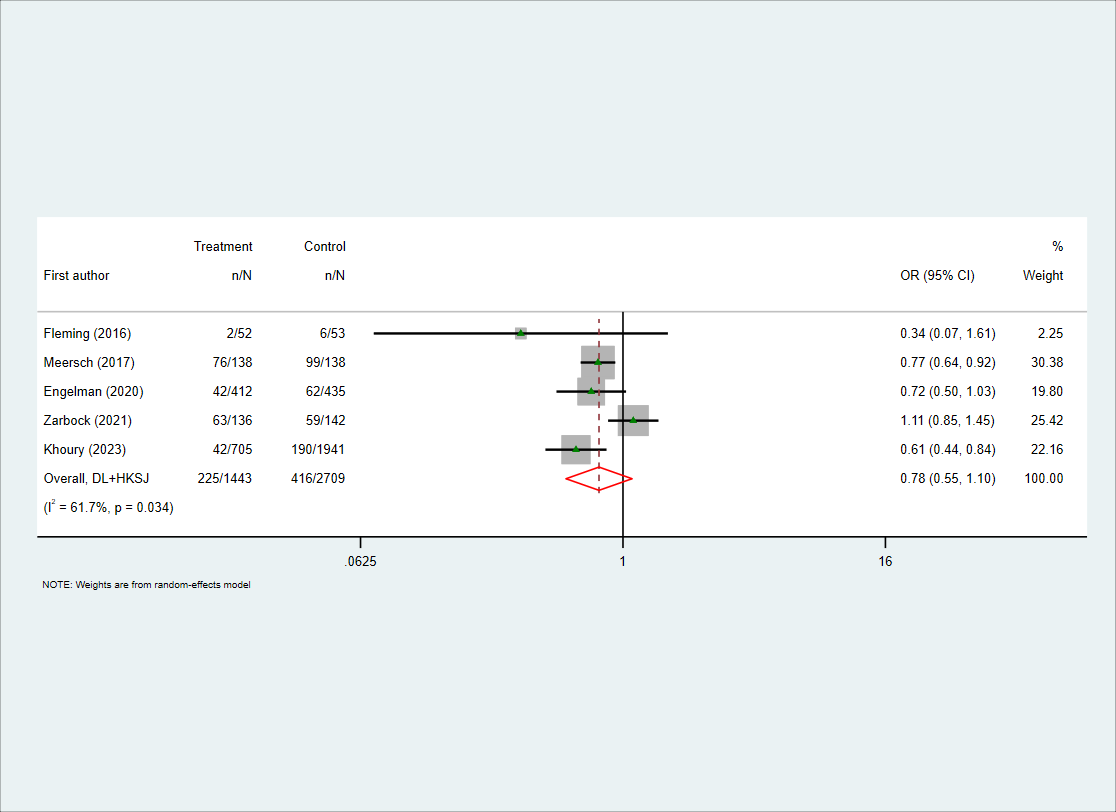


**Figure S7.** Forest plot of the association between receiving care bundle compared to standard care and acute kidney injury in participants with cardiac diseases. The Hartung-Knapp-Sidik-Jonkman (HKSJ) method was applied as a sensitivity analysis. OR: odds ratio; CI: confidence interval. OR: odds ratio; CI: confidence interval.


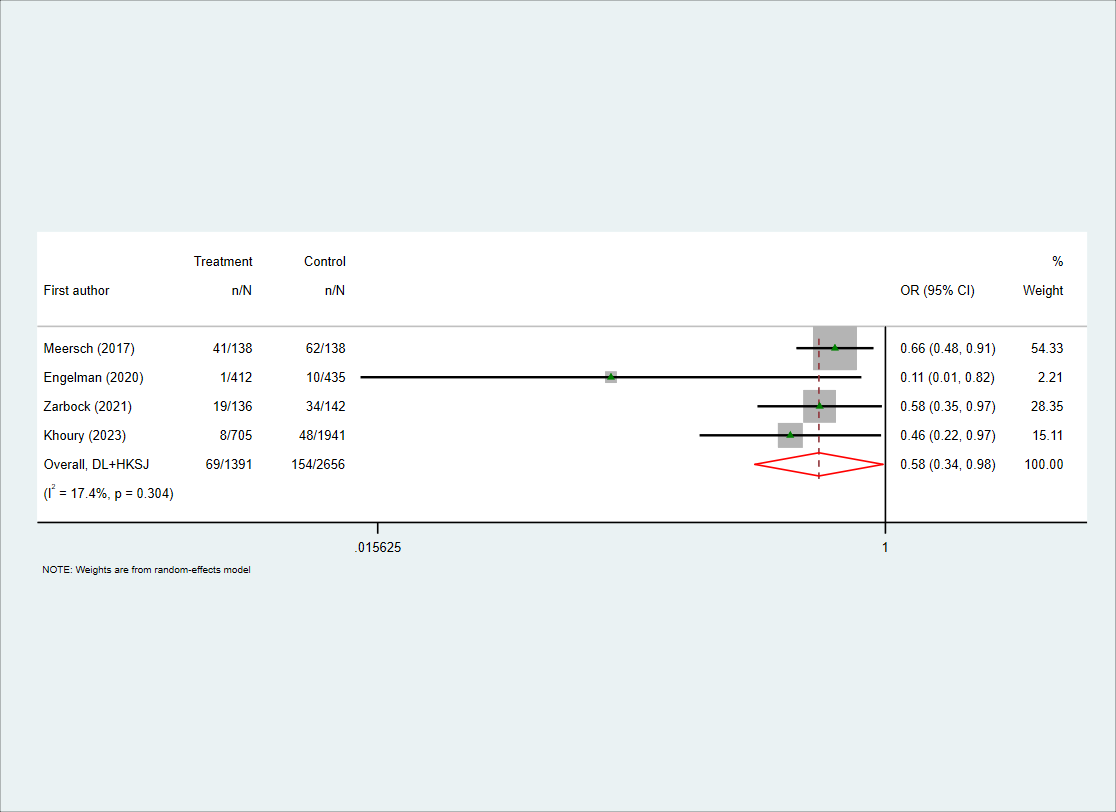


**Figure S8.** Forest plot of the association between receiving care bundle compared to standard care and stage 2 and 3 acute kidney injury in participants with cardiac diseases. The Hartung-Knapp-Sidik-Jonkman (HKSJ) method was applied as a sensitivity analysis. OR: odds ratio; CI: confidence interval.

**References**

1. Fleming IO, Garratt C, Guha R, Desai J, Chaubey S, Wang Y, et al. Aggregation of Marginal Gains in Cardiac Surgery: Feasibility of a Perioperative Care Bundle for Enhanced Recovery in Cardiac Surgical Patients. J Cardiothorac Vasc Anesth. 2016;30(3):665-70.

2. Engelman DT, Crisafi C, Germain M, Greco B, Nathanson BH, Engelman RM, et al. Using urinary biomarkers to reduce acute kidney injury following cardiac surgery. The Journal of Thoracic and Cardiovascular Surgery. 2020;160(5):1235-46.e2.

3. Hoogma DF, Croonen R, Al Tmimi L, Tournoy J, Verbrugghe P, Fieuws S, et al. Association between improved compliance with enhanced recovery after cardiac surgery guidelines and postoperative outcomes: A retrospective study. J Thorac Cardiovasc Surg. 2022.

4. Khoury S, Frydman S, Abu-Katash H, Freund O, Shtark M, Goldiner I, et al. Impact of care bundles on the occurrence and outcomes of acute kidney injury among patients with ST-segment elevation myocardial infarction. J Nephrol. 2023;36(9):2491-7.

5. Massoth C, Kuellmar M, Moncho AP, Suarez SG, Grigoryev E, Ivkin A, et al. Implementation of the Kidney Disease Improving Global Outcomes guidelines for the prevention of acute kidney injury after cardiac surgery <i>An international cohort survey</i>. Eur J Anaesthesiol. 2023;40(6):418-24.

6. Meersch M, Schmidt C, Hoffmeier A, Van Aken H, Wempe C, Gerss J, et al. Prevention of cardiac surgery-associated AKI by implementing the KDIGO guidelines in high risk patients identified by biomarkers: the PrevAKI randomized controlled trial. Intensive Care Med. 2017;43(11):1551-61.

7. Zarbock A, Küllmar M, Ostermann M, Lucchese G, Baig K, Cennamo A, et al. Prevention of Cardiac Surgery-Associated Acute Kidney Injury by Implementing the KDIGO Guidelines in High-Risk Patients Identified by Biomarkers: The PrevAKI-Multicenter Randomized Controlled Trial. Anesth Analg. 2021;133(2):292-302.
